# William F. House: The Father of Neurotology

**DOI:** 10.7759/cureus.69724

**Published:** 2024-09-19

**Authors:** Joshua M Goins, Gauri Mankekar

**Affiliations:** 1 School of Medicine, Louisiana State University Health Sciences Center, Shreveport, USA; 2 Otology and Neurotology, Department of Otolaryngology, Louisiana State University Health Sciences Center, Shreveport, USA

**Keywords:** cochlear implant, historical vignette, medical biographies, medical device innovator, neurotology, pioneer in medicine, william house

## Abstract

William F. House (1923-2012) was an ear, nose, and throat specialist with a particular focus in otology and an inventor, with perhaps his greatest invention being the single-channel cochlear implant. Although heavily criticized at the beginning of his career for developing the single-channel cochlear implant, many individuals would soon benefit from the device that House created. He also revolutionized a new technique for the removal of vestibular schwannomas with assistance from his neurosurgeon colleague, Dr. William Hitselberger. House is considered as the “Father of Neurotology” because of his many advancements in the medical field with refined surgery techniques and life-changing devices.

## Introduction and background

**Figure 1:**
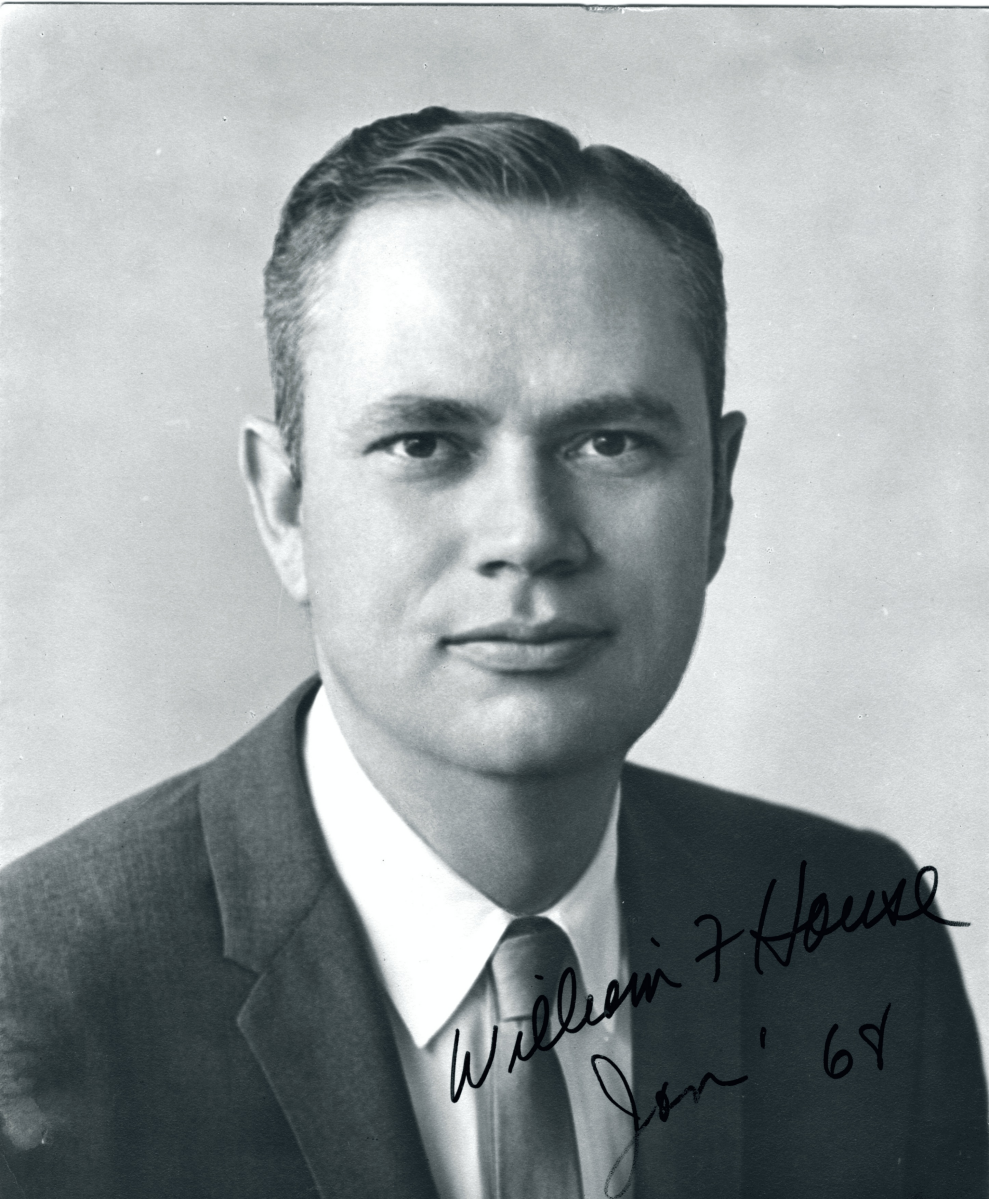
William F. House, MD, 1923-2012 Source: House Ear Institute Archives, Wikimedia Commons, licensed under CC BY-SA 3.0 [5]

The goal of this article is to describe the contributions of William House (1923-2012) to the field of otology. House was an otologist and inventor who devised equipment and procedures to improve many disorders related to the ears with a special focus on conditions involving the inner ear. One of House’s most notable contributions to the medical field was the development and implantation of the first single-channel cochlear implant. Mudry et al. (2013) reported that House read a newspaper article in the late 1950s, describing a patient in Paris who had a wire implanted into the inner ear to stimulate sound conduction [1,2]. House soon entered a partnership with the Doyle brothers with the intent to invent an electrical device that could restore hearing [1]. House was also responsible for the unique utilization of the microscope for middle ear surgery in the United States, inventing the one-handed suction irrigator instrument and developing novel approaches to the removal of vestibular schwannomas alongside neurosurgeon, Dr. William Hitselberger [3]. The one-handed suction irrigator had a finger control on the irrigation part, which helped to facilitate the use of only one hand during ear surgeries without the nurse assistant having to clamp the suction tubing to control the suction. Through these triumphs, House (Figure 1) improved upon existing surgical techniques and invented devices that have made a lasting impact in medicine. One of House’s defining characteristics was his openness to advancing the medical field and truly serving those in need. This was best demonstrated in an email written about Dr. House by an Indian physician after his death in 2012, which stated, “in India, he is a legend, regarded as a saint for his devotion to the betterment of the lives of his patients; giving away his knowledge unselfishly and graciously to all who wanted to learn” [4]. 

## Review

Life and career

Dr. William Fouts House was born in Kansas City, Mo., on December 1, 1923. House originally began his career in dentistry, following in the footsteps of his father, Dr. Milus M. House, and earned his doctorate in dental surgery from the University of California, Berkeley in 1945 [6]. He also met his wife, June Stendahl, around the time of his graduation from dental school. House continued his dental career for a couple of years in the United States Navy performing facial reconstructive surgery. House's time spent in dentistry spurred the idea of creating the one-handed suction irrigator tool [2,4,7]. The development of this instrument greatly aided otologists in performing mastoidectomies by being able to use a drill with one hand while having both the suction and irrigator in the other hand [7]. During his time in the Navy, House decided he wanted to become a Doctor of Medicine. He earned his medical degree from the University of Southern California School of Medicine in 1953 and began his residency to become an ear, nose, and throat specialist [6]. After completing his residency in 1956, he joined the Los Angeles Foundation of Otology (later renamed the House Ear Institute), an establishment founded by House’s older half-brother Dr. Howard House in 1946 [6].

During this time observing Howard, William House gained a special interest in otology. Howard House had focused on otology for several years after he observed physicians in Germany using a microscope for ear surgery and purchased a similar microscope, which William House developed a keen interest in [2]. He would eventually contrive two new methods to remove vestibular schwannomas utilizing the microscope [2,4]. One of House’s most well-known accomplishments occurred on January 9, 1961, when he, alongside neurosurgeon John Doyle, implanted the first single-channel cochlear implant into a patient [1].

House also specialized in treating disorders of the inner ear. It was in this way that House met one of his most famous patients, Captain Alan Shepard of Apollo 14 lunar mission. Shepard struggled with Meniere’s disease, a condition in which there is impaired resorption of endolymph within the inner ear that leads to a triad of intermittent vertigo, hearing loss, and tinnitus. House inserted a small tube into a portion of the inner ear called the endolymphatic sac, resulting in a shunt that transported the endolymph to the subarachnoid space, thus alleviating the swelling of the inner ear [8,9]. Following the procedure, Shepard was able to resume his career at NASA and kept the shunt in place until his death.

House spent most of the remainder of his life trying to perfect the design of the cochlear implant to best help the hearing impaired. He worked at the House Ear Institute in Los Angeles, until his retirement in 2000, when he moved with his wife to Oregon [6]. House passed away on December 7, 2012, at the age of 89 in his Oregon home.

Advancements in neurotology

Dr. House is often referred to as the Father of Neurotology, as he developed many new surgical approaches involving the inner ear and constructed different techniques for the removal of vestibular schwannomas. He became particularly interested in the treatment of vestibular schwannomas in the early 1960s and sought to make changes in that area of medicine. Some of the primary issues surrounding this surgery at that time were the high mortality rate, complete hearing loss, and difficulty in preserving facial nerve function [10]. These are some of the reasons that inspired House to develop the translabyrinthine and middle cranial fossa approach, which was a drastic improvement compared to the previously used suboccipital approach [3,4,10]. Using these novel approaches, House made it possible to operate on vestibular schwannomas and successfully dissect the tumor off the facial nerve, retaining complete function of this nerve [2,10]. He also emphasized the importance of using the microscope to successfully perform these surgeries, thereby improving postoperative outcomes [2,10].

House initially recognized the versatility of the middle cranial fossa approach to removing these tumors while operating on patients with otosclerosis, a disorder involving the stapes bone in the middle ear [11,12]. He theorized that this method could be applied to the removal of vestibular schwannomas and performed the first resection of a vestibular schwannoma utilizing the middle cranial fossa approach alongside John Doyle in 1961. The significance behind this technique was that it led to the possibility of maintaining and, in some cases, improving the hearing in patients affected by these tumors [11]. A main contributor to the success of these surgeries via the middle-fossa approach stemmed from identifying the tumors early, as large tumors were difficult to resect with this approach. The development of computed tomography (CT) in the 1970s and magnetic resonance imaging (MRI) in the 1980s greatly aided in the early identification of these tumors [11]. 

House realized in the preliminary stages of developing these novel approaches that a neurosurgeon would be an essential component of his surgical staff as during this time only neurosurgeons operated on vestibular schwannomas [3]. Dr. Hitselberger completed his neurosurgery residency in 1963 at Henry Ford hospital and then moved to Los Angeles where he met William House. They soon became a dynamic duo [3]. Hitselberger trained under House to learn his techniques for the removal of these tumors. Together they removed over 5,000 vestibular schwannomas and meningiomas with these novel methods [3]. House and Hitselberger passed down these techniques to hundreds of neurotologists around the world, which led to the significant impact on the safety of vestibular schwannoma removal [3,10,13]. Today, the mortality rate for this operation is less than one percent in the US, compared to 40 percent previously [3,10,13]. 

The cochlear implant

**Figure 2:**
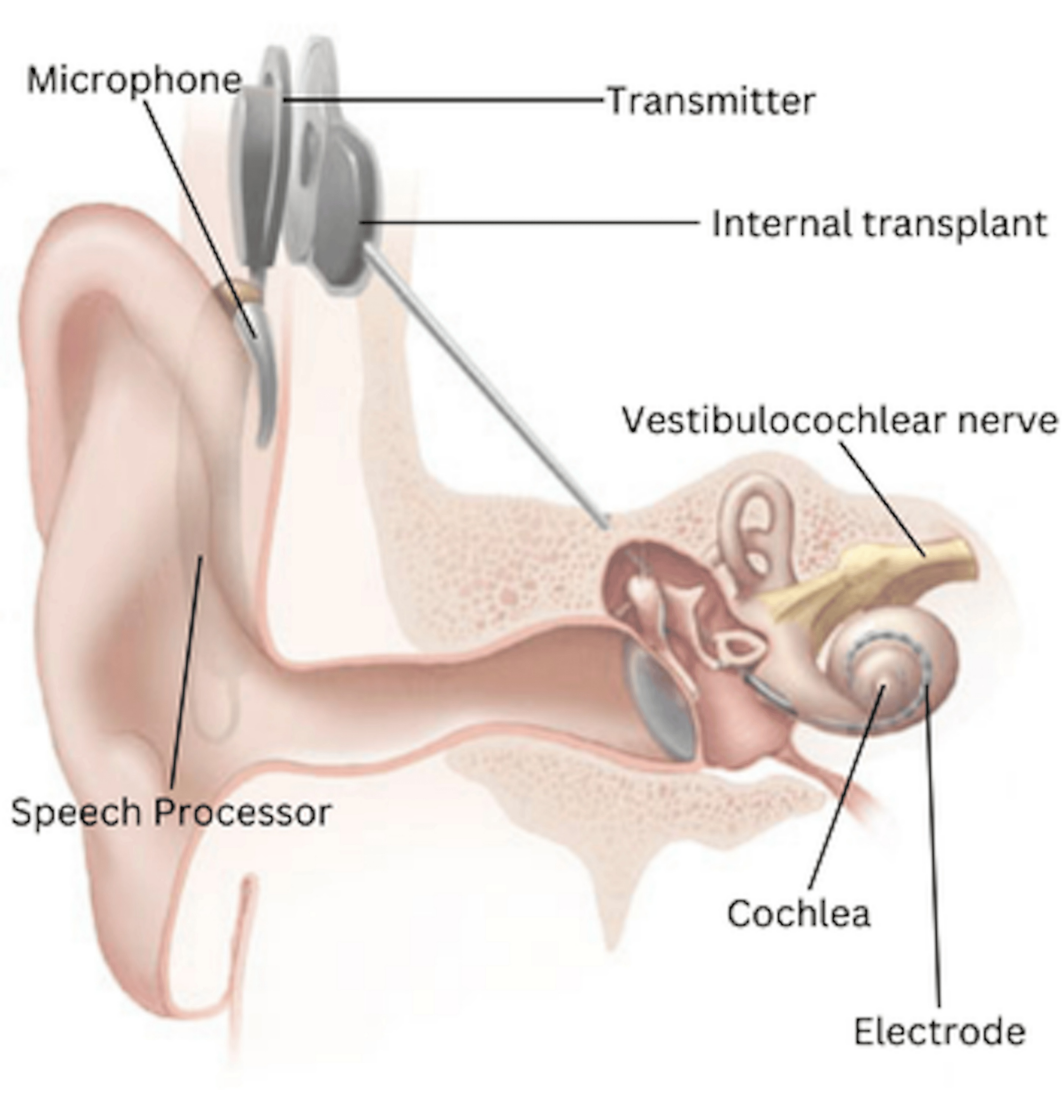
Diagram of a cochlear implant Source: Image courtesy of Wikimedia Commons (public domain), modified from the original [16]

Electrical stimulation of the ear (Figure 2) dates all the way back to the 1700s, but it was not until 1957 that the first semblance of a cochlear implant was inserted by Dr. Charles Eyries, an otolaryngologist, and Andre Djourno, an electrophysicist [1]. They placed an electrode in the internal auditory canal that stimulated the vestibulocochlear nerve. Some do not consider it to be a true cochlear implant as the electrode was not inserted directly into the cochlea [1]. The first cochlear implant with direct insertion was placed by Dr. William House and Dr. John Doyle in 1961 [1,14]. Two patients underwent surgical implantation of a single-wire electrode into the scala tympani in 1961, but one of the patients’ devices had to be removed after just two weeks due to fear of infection [1]. Few reports are available regarding the success of the first cochlear implant; however, it was noted that the patients were able to identify words in small, closed sets [14,15].

**Figure 3:**
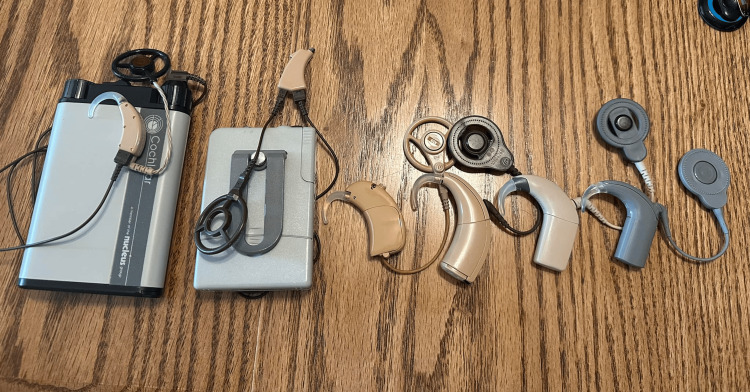
Several generations of cochlear implant processors Source: Image courtesy of Wikimedia Commons (public domain), licensed under CC BY-SA 2.5 [19]

In 1967, House started a new collaboration with engineer Jack Urban [1,14]. Together, they would be the first to create a five-electrode system and later place this device into three patients, the first in 1969, and the other two in 1970 [1,14]. House and Urban published their results on long-term electrode implantation in 1973 and later reviewed these findings with the American Otological Society [1]. During this conference, House realized that the medical community was not yet ready to embrace the idea of cochlear implants because it still had many doubts regarding the efficacy and impact of the devices [1,14,17]. House then enlisted the help of Dr. Miyamoto, a former mentee of House and recent graduate in Neurotology, to persuade the medical community about the success of the cochlear implant by highlighting the extraordinary results in patients with the devices (Figure 3) [14]. Dr. Miyamoto hired several qualified individuals and initiated a multi-centered study to prove that cochlear implants were safe and effective devices [14]. Miyamoto quickly resolved the safety concerns of the procedure; however, demonstrating the devices were effective proved to be a bit more challenging. An independent study conducted by Bilger et al. (1977) altered the perspective about cochlear implants by demonstrating the benefits of the device and helped influence the National Institutes of Health (NIH) to increase funding for cochlear implant research [18]. These studies helped to provide legitimacy to cochlear implantation during a time when the efficacy of cochlear implantation and the ethics of experimentation in this area were questioned.

There were many variations of cochlear implants being invented throughout the 1970s and 1980s, with a special focus on inventing the most effective model [1,14,15]. One of the main differences between House’s cochlear implant and the ones that many other companies/inventors created was that his was a single-channel cochlear implant as opposed to the competitors focusing on multichannel implants. House favored his model of the cochlear implant due to its simpler design, which allowed them to be manufactured for a more affordable price for impoverished individuals [4,17]. The single-channel cochlear implant proved to be successful in improving the hearing in severely hearing disabled and deaf patients [14,15]. The first cochlear implant to be approved by the US Food and Drug Administration (FDA) was the single-channel cochlear implant by 3M/House Ear Institute in 1984 [14,15,17]. This was truly a remarkable accomplishment because the cochlear implant was the first medical device able to restore one of the special senses in humans [4].

Highly effective speech processing strategies, with multiple channels and multiple sites of stimulation in the cochlea, were developed during the 1980s [20]. A consensus statement issued from the 1988 NIH-convened Cochlear Implant Development Conference stated that multi-channel cochlear implants were more effective than single-channel implants due to better word recognition. Today, the multi-channel cochlear implants are the primary devices all over the world [1,15].

The perseverance of Dr. House and hundreds of researchers around the world has led to the development of this revolutionary device that has changed many lives. According to the FDA, by 2010, approximately 219,000 patients had received a cochlear implant [14]. House continued to modify the cochlear implant until his retirement, seeking to develop a less expensive model that could be available to all seeking the device [4].

## Conclusions

Dr. William House excelled in his field of medicine by exhibiting diligence, humility, and ingenuity. It was these personal qualities of House that led to the development of remarkable devices, such as the cochlear implant, and surgical techniques still in use today. The contributions made by House to the field of neurotology remain relevant and continue to inspire many medical professionals around the world.
